# Mesenchymal stromal cells promote B-cell lymphoma in lacrimal glands by inducing immunosuppressive microenvironment

**DOI:** 10.18632/oncotarget.19971

**Published:** 2017-08-07

**Authors:** Min Joung Lee, Se Yeon Park, Jung Hwa Ko, Hyun Ju Lee, Jin Suk Ryu, Jong Woo Park, Sang In Khwarg, Sun-Ok Yoon, Joo Youn Oh

**Affiliations:** ^1^ Department of Ophthalmology, Hallym University Sacred Heart Hospital, Anyang, Korea; ^2^ Laboratory of Ocular Regenerative Medicine and Immunology, Biomedical Research Institute, Seoul National University Hospital, Seoul, Korea; ^3^ Department of Ophthalmology, Seoul National University Hospital, Seoul, Korea; ^4^ R & D Lab, Eutilex Co., Ltd, Seoul, Korea

**Keywords:** apoptosis, B cell lymphoma, lacrimal gland, mesenchymal stromal cells, myeloid-derived suppressor cell

## Abstract

Mesenchymal stromal cells (MSCs) have therapeutic potential for various diseases because of their anti-inflammatory and immunosuppressive properties. However, the immunosuppressive microenvironment allows tumor cells to evade immune surveillance, whereas maintenance of inflammation is required for tumor development and progression. Hence, MSCs may promote or suppress tumors in a context-dependent manner. We here investigated the effects of bone marrow-derived MSCs in a murine model of lacrimal gland B-cell lymphoma. Co-injection of MSCs with B lymphoma cells enhanced tumor growth in lacrimal glands without long-term engraftment. Of note, MSCs induced greater infiltration of immune and immune-regulatory cells near tumor: CD4^+^ cells, CD11b^+^ cells, CD4^+^Foxp3^+^ regulatory T cells and CD11b^+^Ly6C^+^Ly6G^−^ myeloid-derived suppressor cells. Concurrently, there was up-regulation of immune-related molecules including TNF-α, IL-1β, TGF-β1, and arginase in glands treated with MSCs. Apoptosis in tumor was less severe in mice treated with MSCs compared to those without MSCs; however, MSCs did not directly inhibit apoptosis of B lymphoma cells in an *in vitro* co-culture. Together, data demonstrate that MSCs create immunosuppressive milieu by recruiting regulatory immune cells and promote B-cell lymphoma growth in lacrimal glands.

## INTRODUCTION

The anti-inflammatory and immunosuppressive properties of mesenchymal stromal cells (MSCs) provide a major advantage for their use in cell-based therapies for a variety of immune-mediated diseases. However, the actions of MSCs to regulate immune response might bring a favorable or unfavorable outcome in patients in the context of tumor development. Since inflammation is an important risk factor for tumor initiation and progression, MSCs may suppress tumors by mitigating tumor-associated inflammation. On the other side, MSCs may promote tumors by creating the immunosuppressive microenvironment required for tumor cells to evade immune surveillance and by repressing anti-tumor immune response. Previous studies have exhibited conflicting results regarding effects of MSCs on tumors, which largely vary by type, origin, or site of tumors. Therefore, it is important to note the double-edged effects of MSCs on tumor development for MSC-based therapies to be safe and successful in various immune-mediated indications.

Our group recently observed that local injection of bone marrow-derived MSCs into lacrimal glands protected the ocular surface in mice with dry eye disease (DED) by suppressing CD4^+^ T cell-induced inflammation [1]. Since inflammation in lacrimal glands plays an essential role in the pathogenesis of DED, the MSC-based immune modulation would offer a novel therapeutic opportunity for treating severe DED in patients with Sjögren’s syndrome (SS) or ocular graft-versus-host disease (GVHD). Importantly, inflammation is involved in the pathogenesis of B cell lymphomas in lacrimal glands. For example, B-cell non-Hodgkin lymphoma (NHL) is the major complication of SS [2-4]. The risk of developing NHL in SS patients was estimated to be 44 times greater than that in normal population [4]. Therefore, it is possible that the MSC injection to treat lacrimal gland inflammation in DED patients with SS or ocular GVHD might alter the immune microenvironment in favor of or against the tumor development.

In the present study, we established a syngeneic lacrimal gland B-cell lymphoma model in immuno-competent mice and investigated the effects of MSCs on immune cells and tumor development in lacrimal glands. We injected A20 cells into lacrimal glands to induce diffuse large B-cell lymphoma (DLBCL), because B-cell lymphoma is the most common NHL accounting for 30∼40% of all NHLs and DLBCL is the most common high-grade ocular adnexal lymphoma associated with the 5-year overall survival rate being 60% [5, 6]. Our data demonstrate that a co- injection of MSCs and A20 cells into lacrimal glands promotes B-cell lymphoma growth and increased infiltration of various immune and immune-regulatory cells including CD4^+^ cells, CD11b^+^ cells, Foxp3^+^ regulatory T cells (Tregs), or CD11b^+^Ly6C^+^Ly6G^−^myeloid-derived suppressor cells (MDSCs).

## RESULTS

### Establishment of lacrimal gland B-cell lymphoma model and characterization of tumor microenvironment

**Figure 1:**
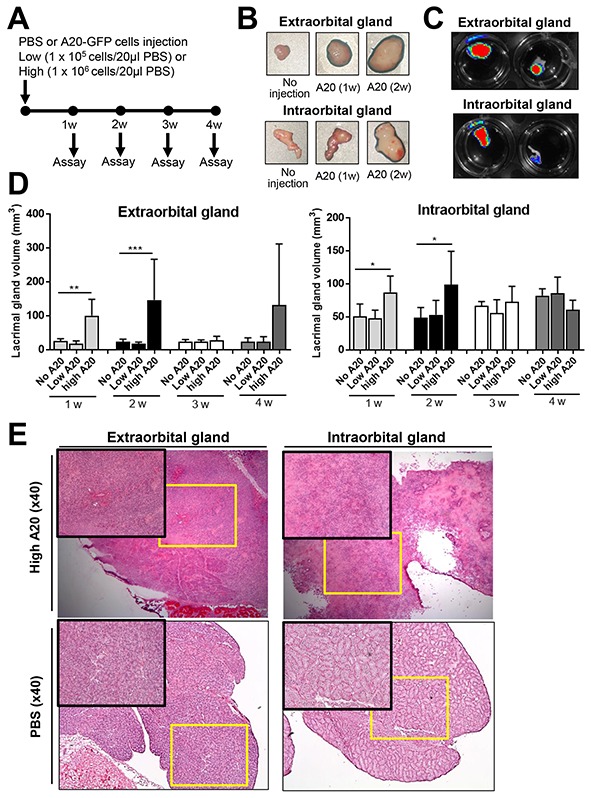
Establishment of B-cell lymphoma model in lacrimal glands **(A)** Schema of experiments. The GFP-labelled A20 B lymphoma cells (A20-GFP), either 1 × 10^5^ (low A20) or 1 × 10^6^ A20 cells (high A20), were injected into extra- and intraorbital lacrimal glands of BALB/c mice. PBS was injected as control (No A20). The glands were assayed every week until 4 weeks post-injection. **(B)** Serial gross images of lacrimal glands after injection of 1 × 10^6^ A20-GFP (high A20). **(C)** GFP fluorescent imaging of lacrimal glands at 2 weeks after injection of 1 × 10^6^ A20-GFP. **(D)** The volume measurements of lacrimal glands (mean + SD) at 1, 2, 3, and 4 weeks after injection of 1 × 10^5^ or 1 × 10^6^ A20 cells. The data represent three independent sets of experiments (four mice per group in each experiment). * *p* < 0.05, ** *p* < 0.01. **(E)** Representative hematoxylin-eosin-stained sections of extraorbital and intraorbital glands at 2 weeks after 1 × 10^6^ A20 cells or PBS injection. Original magnification × 40.

To create a lacrimal gland B-cell lymphoma model, we injected 1 × 10^5^ or 1 × 10^6^ GFP-transduced B lymphoma cells (A20, H-2^d^) into extraorbital and intraorbital lacrimal glands in immunocompetent syngeneic BALB/c mice (H-2^d^). The mouse has two types of lacrimal glands, one of which is located in an intraorbital space and the other in the extraorbital space under the skin anterior to the ear. Because the intra- and extraorbital microenvironments are different, we injected the tumor cells into both glands and investigated the tumor growth. The same volume of Phosphate-buffered solution (PBS) was injected in the control group. At 1, 2, 3, and 4 weeks post-injection, mice were sacrificed and lacrimal glands collected for evaluation of volume, GFP immunofluorescence, and histology (Figure 1A). The mice showed no signs of illness in all groups and continued to gain weight without difference between groups (Supplementary Figure 1A).

There were significant increases in the volume of the extra- and intraorbital lacrimal glands at 1 and 2 weeks after intra-gland injection of 1 × 10^6^ A20 cells (Figure 1B-1D). The fluorescent imaging of the whole glands showed the presence of GFP-positive mass in the extra- and intraorbital glands at 1 and 2 weeks after 1 × 10^6^ A20 cell injection, indicating proliferation of the injected A20 cells and formation of B-cell lymphoma (Figure 1C). In 6 out of 14 extraorbital glands, tumors continued growing to form an enormous mass until 4 weeks (Supplementary Figure 2). Injection of 1 × 10^5^ A20 cells did not induce any significant volume changes in any of extra- or intraorbital lacrimal glands at any time-points (Figure 1D).

**Figure 2:**
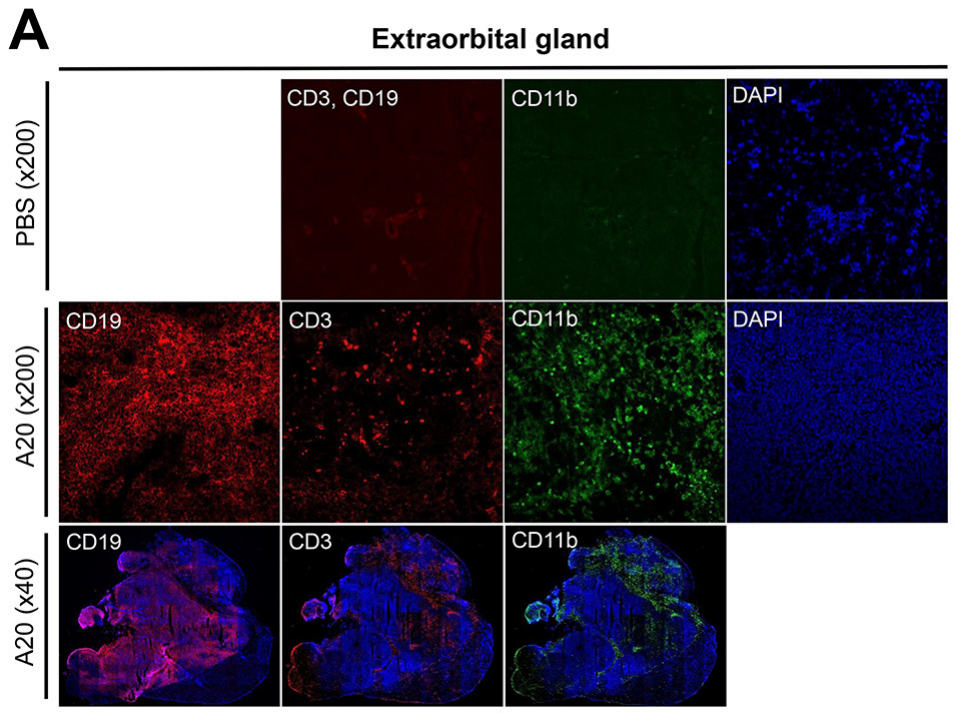

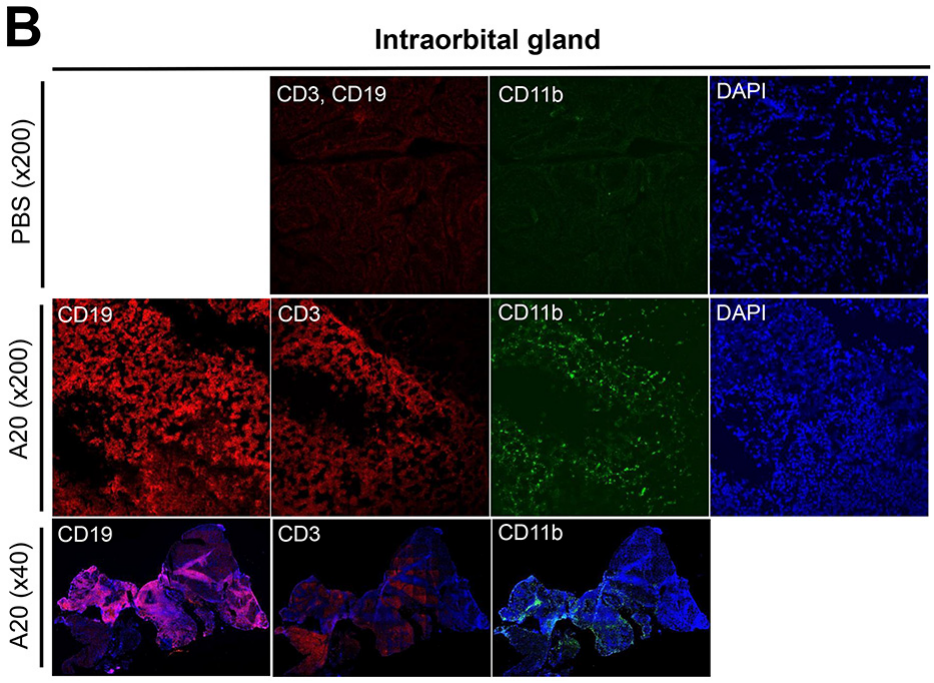
Immunohistochemical characterization of B-cell lymphoma model in lacrimal glands Immunohistochemical staining for CD19, CD3, and CD11b of extraorbital **(A)** and intraorbital glands **(B)** at 2 weeks after 1 × 10^6^ A20 B lymphoma cell or PBS injection. Shown were tumor masses composed of CD19^+^ cells which were surrounded by CD3^+^ and CD11b^+^ cells in A20 cell-injected glands.

Hematoxylin-eosin staining revealed extensive infiltration of tumor cells, severe destruction of normal acinar and ductal structure, and accompanying necrosis and blood vessels in the extra- and intraorbital glands at 1 and 2 weeks after 1 × 10^6^ A20 cell injection (Figure 1E). Immunohistochemical staining for CD19 showed that the majority of tumor cells infiltrating lacrimal glands were B cells (Figure 2). To evaluate the composition and spatial arrangement of immune cells that constitute the tumor microenvironment (TME), we immunostained the glands for CD3 and CD11b because T cells and monocytes/macrophages are key cellular components in TME of B-cell lymphoma [7-9]. Numerous CD3^+^ cells and CD11b^+^ cells were detected in close contact with CD19^+^ tumor cells in extra- and intraorbital lacrimal glands (Figure 2, Supplementary Figure 3).

Therefore, these data demonstrate that B-cell lymphoma developed in lacrimal glands at 1 and 2 weeks following an intra-gland injection of A20 B lymphoma cells, and a number of CD3^+^ and CD11b^+^ cells infiltrated the tumor.

### MSCs promote B lymphoma cell growth in lacrimal glands

**Figure 3:**
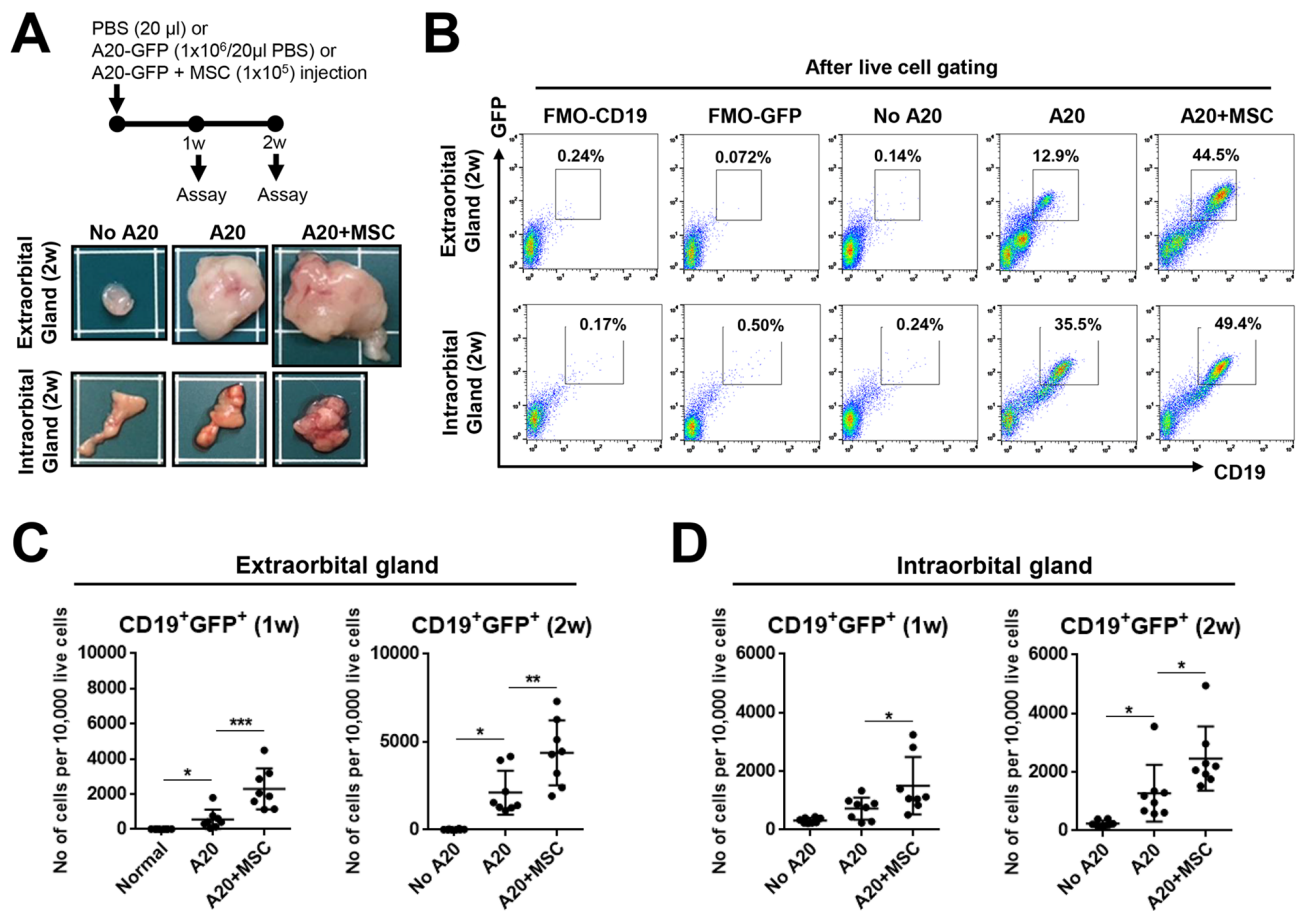
MSCs promote growth of lacrimal gland B-cell lymphoma **(A)** Experimental scheme and representative photographs of extraorbital and intraorbital glands at 2 weeks after 1 × 10^6^ A20 B lymphoma cell injection or A20+MSC co-injection. PBS was injected as negative control (No A20). **(B-D)** Representative and quantitative flow cytometry results for CD19^+^GFP^+^ cells in extraorbital (C) and intraorbital lacrimal glands (D) at 1 and 2 weeks after A20 or A20+MSC injection. FMO (fluorescence minus one) control per each antibody was used as gating control, and the analysis was performed after excluding dead cells with FVD (Fixable Viability Dye) staining. Dot indicates a single animal, and the bar represents the mean ± SD. * *p* < 0.05, ** *p* < 0.01, *** *p* < 0.001.

To investigate the effects of MSCs on lacrimal gland B-cell lymphoma, we mixed 1 × 10^6^ GFP-labelled A20 cells with 1 × 10^5^ bone marrow (BM)-derived human MSCs and injected into extra- and intraorbital lacrimal glands of BALB/c mice. For comparison, either 1 × 10^6^ A20 cells alone or the same volume of PBS was injected into the glands of control mice. There were no differences in body weight between groups at all time-points (Supplementary Figure 1B). To quantitatively analyze the tumor, we sacrificed the mice, extracted extra- and intraorbital lacrimal glands, and isolated cells at 1 and 2 weeks post-injection. The cells were evaluated for the expression of CD19 and GFP using flow cytometry (Figure 3).

Similar to the gland volume results (Figure 1D), tumor mass was grossly observed in glands (Figure 3A). Flow cytometric analysis of live cells revealed that the number of CD19^+^GFP^+^ cells was increased in the extra- and intraorbital glands at 1 and 2 weeks following A20 cell injection, compared to PBS-injected controls (Figure 3B-3D). Because most of CD19^+^ cells were positive for GFP (Figure 3B), CD19^+^ cells in lacrimal glands were B-lymphoma cells which originated from injected A20 cells.

Of note were the findings that MSC co-injection markedly increased the gland size and tumor formation (Figure 3A). Consistent with these, the number of CD19^+^GFP^+^ cells was significantly higher at both 1 and 2 weeks in extra- and intraorbital lacrimal glands receiving A20 cells and MSCs, compared to those receiving A20 cells alone (Figure 3B-3D).

### MSCs increase regulatory T cells and myeloid-derived suppressor cells in tumor-bearing lacrimal glands

Since we observed that CD3^+^ T cells and CD11b^+^ monocytes/macrophages are abundantly present in the tumor tissues (Figure 2, Supplementary Figure 3), we further characterized the cell subsets infiltrating the lymphoma and investigated the effects of MSCs on the subsets. Single cell suspension was prepared from lacrimal gland tumors at 1 and 2 weeks after A20 or A20+MSC injection and analyzed for immune cell subsets by flow cytometry.

**Figure 4:**
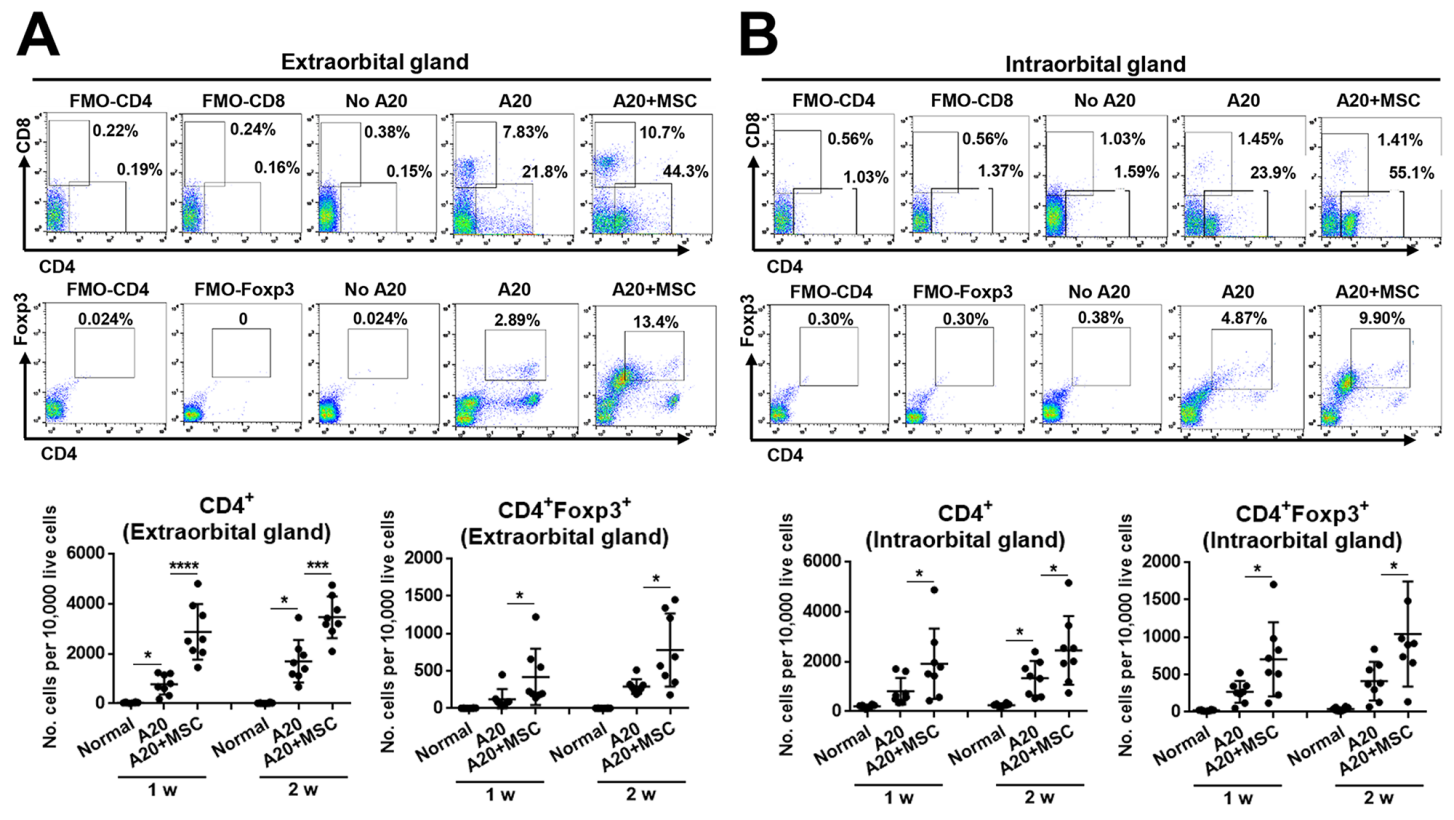
MSCs increase CD4^+^ and CD4^+^ Foxp3^+^ cells in lacrimal glands bearing B-cell lymphoma **(A, B)** Representative and quantitative flow cytometry results for CD4^+^, CD8^+^, CD4^+^Foxp3^+^ regulatory T cells in extraorbital (A) and intraorbital lacrimal glands (B) at 1 and 2 weeks after either 1 × 10^6^ A20 cell injection or A20+MSC co-injection. PBS was injected in control group (No A20). FMO (fluorescence minus one) control per each antibody was used as gating control, and the analysis was performed after excluding dead cells with FVD (Fixable Viability Dye) staining. Dot indicates a single animal, and the bar indicates the mean ± SD. * *p* < 0.05, *** *p* < 0.001, **** *p* < 0.0001.

CD4^+^ cells were the most prevalent cells that infiltrated both extra- and intraorbital lacrimal glands bearing tumors, while CD8^+^ cells were increased in the extraorbital glands but not in intraorbital glands (Figure 4). The number of CD4^+^ cells was significantly higher in the extra- and intraorbital glands receiving MSC co-injection, compared to those receiving A20 cells alone (Figure 4). Similarly, CD4^+^Foxp3^+^ regulatory T cells (Tregs) were also elevated in both extra- and intraorbital glands by MSC injection (Figure 4).

**Figure 5:**
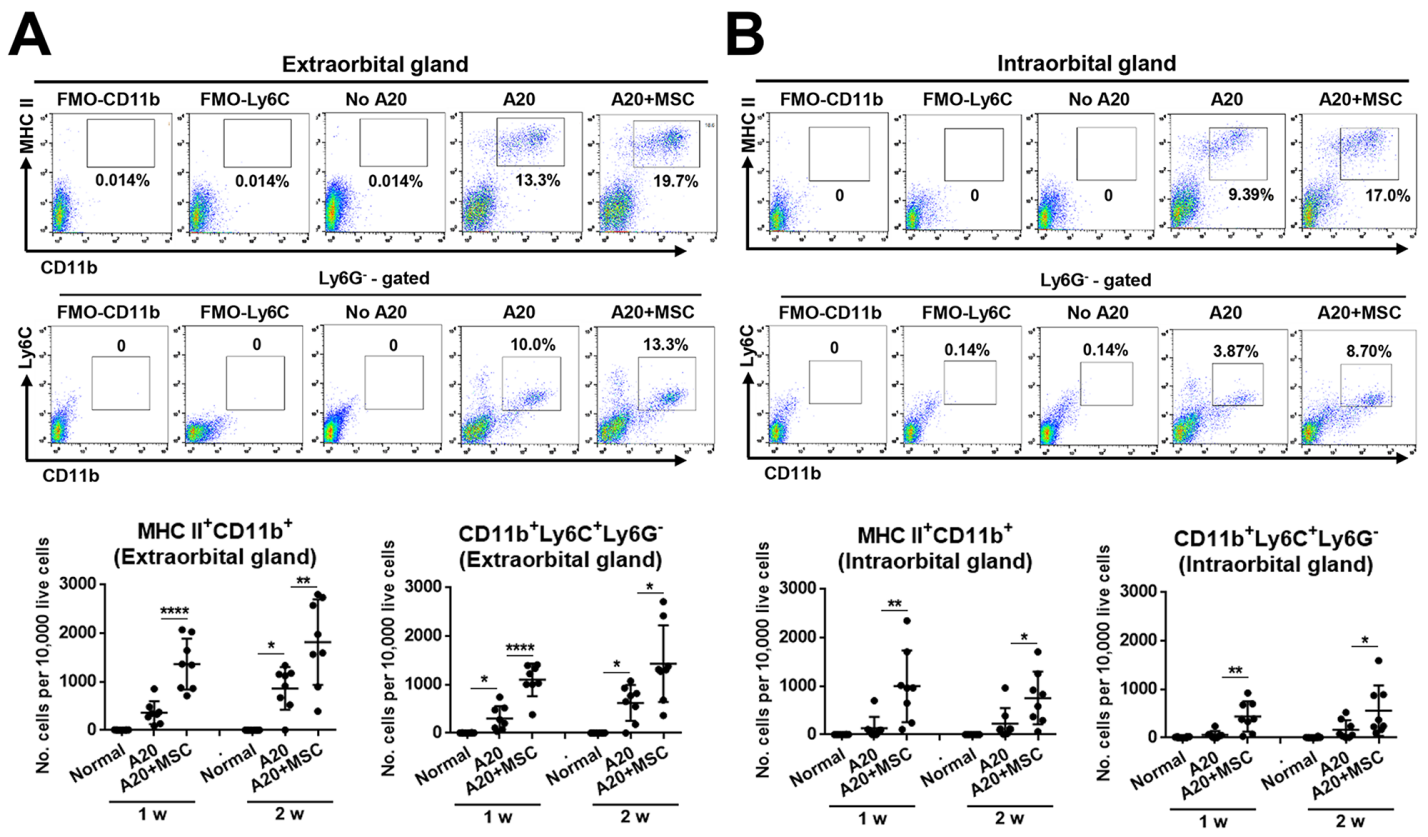
MSCs increase MHC II^+^CD11b^+^ and CD11b^+^Ly6C^+^Ly6G^−^ cells in lacrimal glands bearing B-cell lymphoma **(A, B)** Representative and quantitative flow cytometry results for MHC class II^+^CD11b^+^ and CD11b^+^Ly6C^+^Ly6G^−^ cells in extraorbital (A) and intraorbital lacrimal glands (B) at 1 and 2 weeks after either 1 × 10^6^ A20 cell injection or A20+MSC co-injection. No A20 group indicates control group with PBS injection. FMO (fluorescence minus one) control per each antibody was used as gating control, and the analysis was performed after excluding dead cells with FVD (Fixable Viability Dye) staining. Dot indicates a single animal, and the bar indicates the mean ± SD. * *p* < 0.05, ** *p* < 0.01, **** *p* < 0.0001.

As for monocytes/macrophages, there was a marked increase in MHC II^+^CD11b^+^ cells in glands bearing lymphoma (Figure 5), which is consistent with our immunohistochemistry data (Figure 2). Most prominent was the increase in CD11b^+^Ly6C^+^Ly6G^−^ cells (Figure 5) which are monocytic MDSCs [10]. Notably, there was greater expansion of both MHC II^+^CD11b^+^ cells and CD11b^+^Ly6C^+^Ly6G^−^ MDSCs in the extra- and intraorbital glands receiving co-injection of A20 and MSCs, compared to those receiving A20 cells alone (Figure 5). Similar effects were observed with tumor-infiltrating dendritic cells (DCs). The number of MHC II^+^CD11c^+^ DCs was increased in the glands by MSC co-implantation, and most of DCs were CD40^+^CD11c^+^ cells (Supplementary Figure 4).

### Cytokine profile in lacrimal glands containing tumor

**Figure 6:**
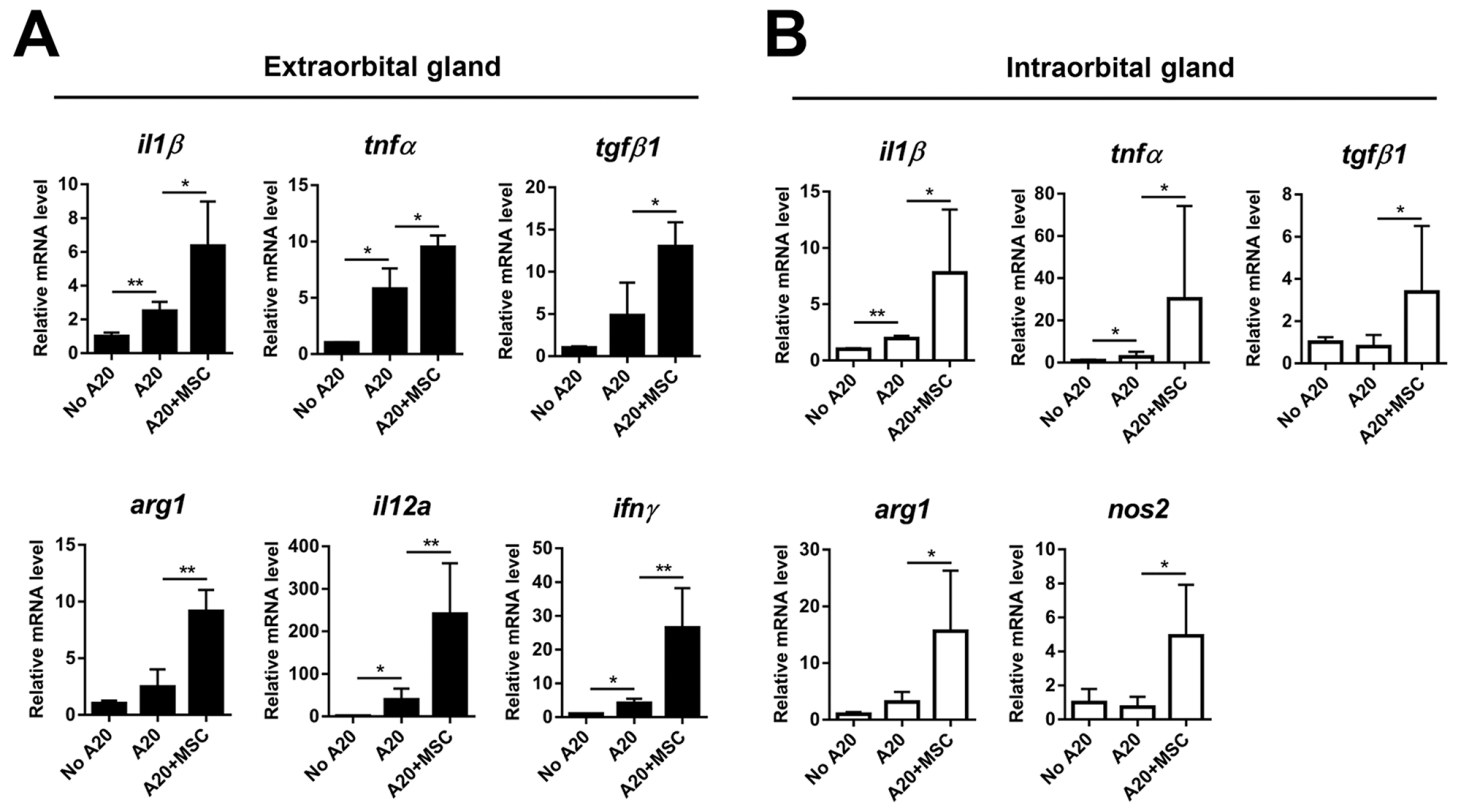
Molecular assays of lacrimal glands bearing B-cell lymphoma with or without MSCs Real-time RT PCR analysis for inflammatory and immune-regulatory molecules in extraorbital **(A)** and intraorbital glands **(B)** at 2 weeks after 1 × 10^6^ A20 injection or A20+MSC co-injection. *Arg1*: arginase, *Nos2*: inducible nitric oxide synthase. Data (mean + SD) are presented as the fold changes relative to PBS-injected controls (No A20). * *p* < 0.05, ** *p* < 0.01.

We next evaluated the levels of immune-related factors that are known to be involved in the pathogenesis of lymphoma (Figure 6) [8, 9]. Real-time RT PCR assay showed that the levels of TNF-α and IL-1β, pro-inflammatory cytokines known as important factors for tumorigenesis [9], were elevated in extra- and intraorbital lacrimal glands receiving A20 cells, reflecting an inflammatory milieu in tumor-bearing glands. TNF-α is produced by multiple cell types including macrophages, B cells, T cells, epithelial cells or fibroblasts, while IL-1β is secreted mainly by innate immune cells such as macrophages and DCs [9]. MSC co-injection markedly upregulated the levels of TNF-α and IL-1β in the glands. The level of TGF-β1, which is an immunosuppressive cytokine in TME produced by T cells, B cells or macrophages [9], was also increased by MSCs. In addition, we evaluated the level of arginase (Arg1) because MDSCs produce arginase to deplete arginine, an essential amino acid for T-cell activity and induce T-cell tolerance [10-13]. Consistent with the increase of MDSCs in MSC-treated glands (Figure 5), the level of arginase was highly increased in both extra- and intraorbital lacrimal glands by MSC co-injection. Similarly, IL-12 and IFN-γ were up-regulated in the extraorbital gland receiving A20 cells and further increased by MSC co-injection, but they were not expressed in intraorbital glands. IL-12 is largely secreted by macrophages and DCs, and IFN-γ produced by T cells, B cells, macrophages and DCs [9]. The level of inducible nitric oxide synthase (Nos2), a molecule mediating T cell suppression by MDSCs [10, 11], was increased in intraorbital glands by MSCs, but not expressed in extraorbital glands. No IL-2, -4, or -10 was detected in any of the glands evaluated (data not shown). The increased levels of TNF-α, IL-12 and IFN-γ and undetectable levels of IL-4 and IL-10 suggest that TME in lacrimal gland B-cell lymphoma was polarized toward Th1 pattern, which is similar to what were previously observed with an intraocular B-lymphoma [8].

### Apoptosis is reduced in tumors of lacrimal glands treated with MSCs

**Figure 7:**
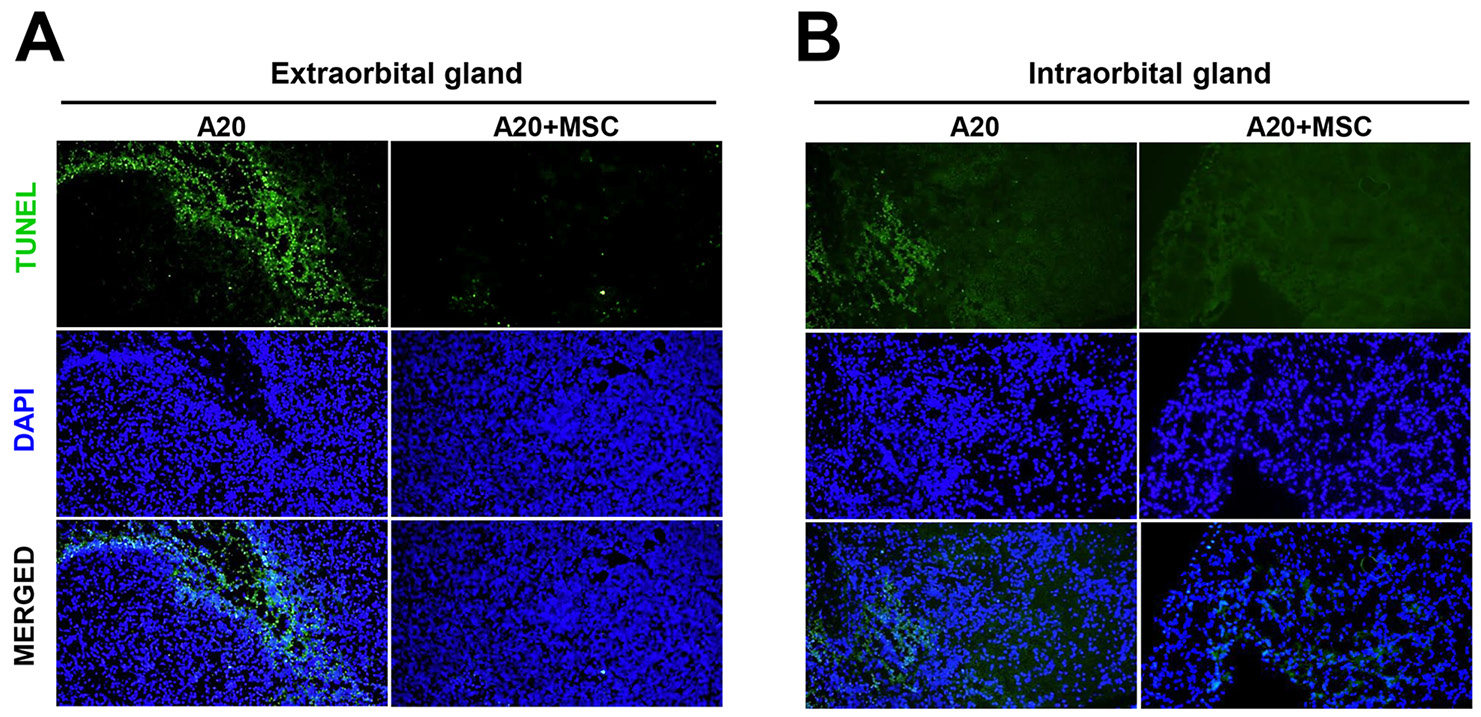
MSCs reduce tumor apoptosis in lacrimal glands bearing B-cell lymphoma *in vivo* Representative pictures of TUNEL (terminal deoxynucleotidyl transferase dUTP nick end labeling) staining of extraorbital **(A)** and intraorbital glands **(B)** 2 weeks after 1 × 10^6^ A20 injection or A20+MSC. DAPI was used for nuclear counter-staining. Original magnification × 200.

We next sought to determine whether MSCs exacerbate B-cell lymphoma by inhibiting tumor cell apoptosis. TUNEL (terminal deoxynucleotidyl transferase dUTP nick end labeling) staining of the glands revealed that multiple TUNEL^+^ apoptotic cells were present in tumors, but there were fewer TUNEL^+^ cells in those treated with MSCs (Figure 7). The similar results were obtained with both extra- and intraorbital lacrimal glands.

**Figure 8:**
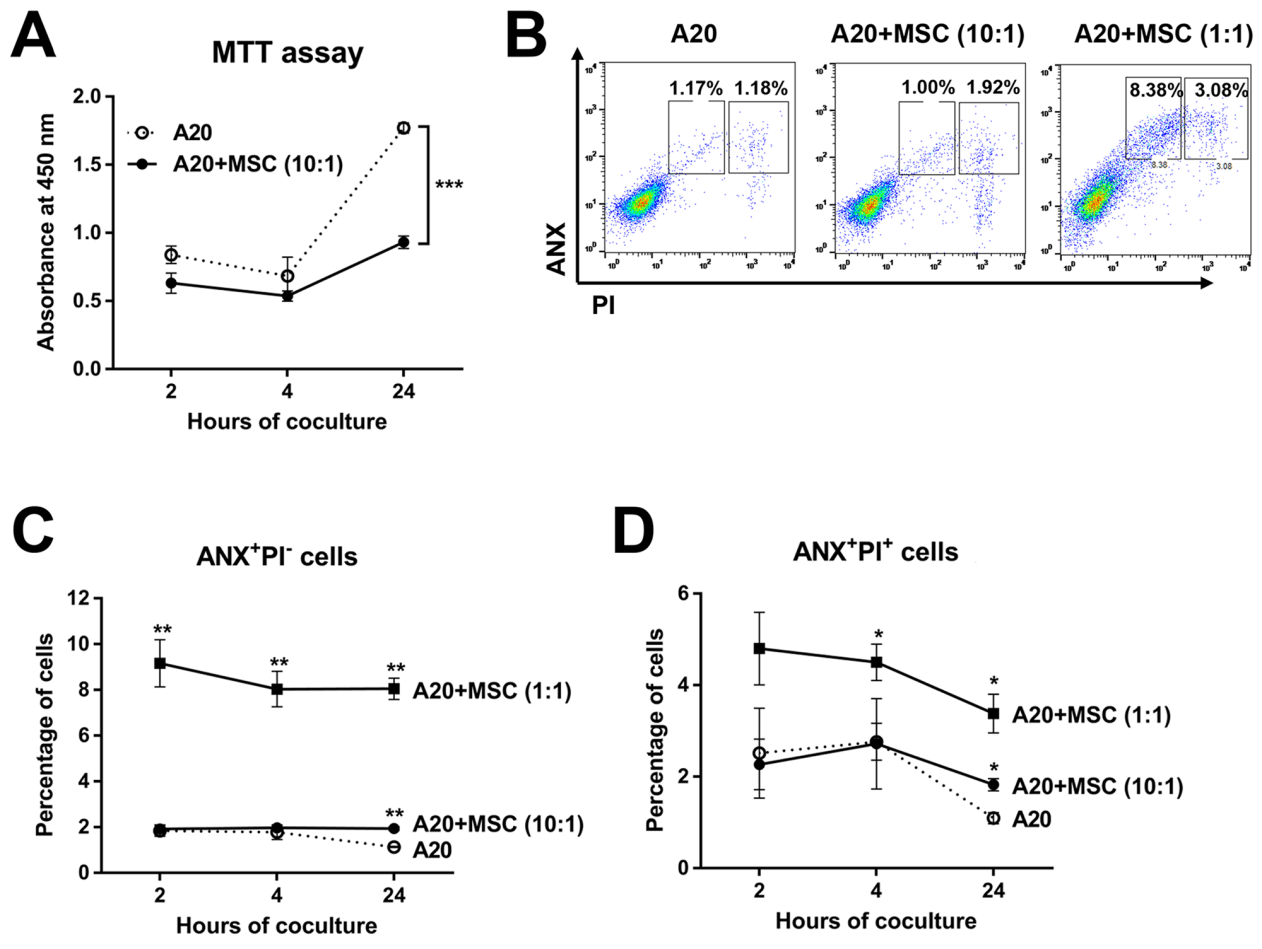
Direct effects of MSCs on survival and apoptosis of A20 B lymphoma cells *in vitro* **(A)** The MTT assay of A20 cells at 2, 4, and 24 hours of co-culture with MSCs (A20 to MSC ratio = 10:1). **(B, C, D)** Flow cytometric analysis for early apoptotic (ANX^+^PI^−^) and late apoptotic (ANX^+^PI^+^) cells in A20 cells after 2, 4, and 24 hours of co-culture with MSCs (A20 to MSC ratio = 10:1 or 1:1). Data are presented as mean ± SD from three independent sets of experiments (three samples in each group per a set). Asterisks indicate the statistical significance between A20 and A20+MSC (1:1 or 10:1) at each time-point. * *p* < 0.05, ** *p* < 0.01, *** *p* < 0.001.

To test whether the apoptosis reduction in tumors might be due to the direct anti-apoptotic effect of MSCs on tumor cells, we next co-cultured A20 cells and MSCs *in vitro* and measured the survival and apoptosis of A20 cells. Contrary to the *in vivo* findings, the viability of lymphoma cells was markedly inhibited by MSCs as suggested by the MTT (3-(4,5-dimethylthiazol-2-yl)-2,5-diphenyl tetrazolium bromide) assay (Figure 8A). Flow cytometric analysis of tumor cells after Annexin V (ANX) and propidium iodide (PI) staining revealed that MSCs significantly induced both early (ANX^+^PI^−^ cells) and late apoptosis (ANX^+^PI^+^ cells) in A20 lymphoma cells in a dose-dependent manner (Figure 8B-8D). These data indicate that the effects of MSCs in reducing apoptosis and promoting tumor were not due to direct effects of MSCs on tumor cell survival.

### MSCs do not engraft lacrimal glands

It was previously demonstrated that exogenous administration of MSCs exerts their effects without long-term engraftment by activating endogenous immune regulatory system of the recipient [14]. To evaluate the engraftment of MSCs in lacrimal glands after a concomitant injection with A20 lymphoma cells, we carried out immunohistochemical staining for human-specific mitochondria and human nuclear antigen because MSCs we injected were of human origin. Results showed that there were no MSCs in either extraorbital or intraorbital lacrimal gland at 1 and 2 weeks post-injection (Supplementary Figure 5), which is the same as our previous observation with MSC injection into lacrimal glands in DED mice [1].

## DISCUSSION

Our data demonstrate that a local injection of MSCs promotes B-cell lymphoma in lacrimal glands without the long-term engraftment of the cells. The tumor-promoting effects of MSCs were accompanied by a marked increase in CD4^+^ cells, CD11b^+^ cells, CD4^+^Foxp3^+^ Tregs, and CD11b^+^Ly6C^+^Ly6G^−^ MDSCs.

B lymphoma cells utilize multiple mechanisms for immune evasion including alterations in surface molecules to reduce immunogenicity, direct interactions with immune cells that dampen their function, and recruitment of cells that induce immunosuppression [7]. Most predominant among the immune cells recruited by B-cell lymphoma are Tregs [7, 15], which induce host tolerance to tumor-specific antigens of malignant B cells [16]. In addition, an increasing number of studies suggest that MDSCs play an important role during lymphoma progression by suppressing T cells and expanding Tregs [17-20]. MDSCs include two distinct subtypes: CD11b^+^Ly6C^hi^Ly6G^−^ monocytic MDSCs and CD11b^+^Ly6C^lo^Ly6G^+^ granulocytic MDSCs [10]. Recent studies demonstrate that the number of monocytic MDSCs was correlated with the circulating Treg number, prognosis and survival of patients with diffuse large B-cell lymphoma (DLBCL) [18, 20, 21]. In our study, an exogenous administration of MSCs significantly increased the infiltration of Tregs and monocytic MDSCs in the A20 B-cell lymphoma model of the lacrimal gland, a model for DLBCL in mice. The results suggest a possibility that Tregs and MDSCs might be one of the cellular players mediating the tumor-promoting action of MSCs. Further studies to deplete Tregs and MDSCs using blocking antibodies or knockout mice would be necessary to confirm the role of these regulatory cells in mediating the MSC action in lymphoma progression.

Similar observation was made by Ren et al. in mice bearing p53 mutations that develop spontaneous lymphoma [22]. They showed that BM-derived murine MSCs promoted tumor growth when injected along with lymphoma cells into the hind legs of immunocompetent syngeneic mice. The greater effects on lymphoma growth were obtained with lymphoma-derived MSCs. They further revealed that the tumor-promoting effects of BM- or lymphoma-derived MSCs were due to CCR2-mediated recruitment of CD11b^+^Ly6C^+^ monocytes. In contrast, Song et al. reported that an intravenous co-infusion of murine BM-MSCs and A20 cells decreased the incidence of lymphoma and ameliorated acute GVHD in a murine model of allogeneic BM transplantation [23]. Therefore, it is possible that MSCs may be beneficial or detrimental depending on the type and location of tumor, origin and donor of MSCs, or method of cell administration (route or timing), accounting for largely conflicting results between studies.

Despite their potential of tumor-supporting role under certain conditions, MSCs have emerged as a promising candidate for anti-tumor therapy. MSCs can be engineered to express pro-apoptotic factors for tumors or utilized to deliver anti-cancer agents into the tumor owing to their remarkable homing ability to tumor tissues. For instance, MSCs overexpressing TNF-related apoptosis inducing ligand have been reported to inhibit tumor growth and metastasis in animal models for mesothelioma, sarcoma, and breast cancer [24-26].

In this study, we used a model of orthotopic transplantation of syngeneic tumor cells into lacrimal glands in immunocompetent mice. Although models of tumor xenograft in immunodeficient mice have been widely used for studying cancers, the use of immunocompetent mice would help understand how MSCs interact with both adaptive and innate immune cells and affect tumor development and progression.

Considering the lymphoma-promoting potential of MSCs shown in our study, caution should be exercised when using local administration of MSCs for treatment of ocular diseases such as DED and SS [1]. A better understanding of the mechanisms underlying tumor support or suppression by MSCs may help increase the utilization of MSCs in regenerative medicine without risk of promoting pre-existing tumor cell growth.

## MATERIALS AND METHODS

### Preparation of A20 cells and MSCs

Murine A20 B lymphoma cell line (H-2^d^; diffuse large B-cell lymphoma cell line) [27, 28], which is a B lymphoma cell line obtained from a spontaneous reticulum neoplasm in old BALB/c mice, was obtained from the American Type Culture Collection (ATCC, Rockville, MD) and transduced with the green fluorescent protein (GFP)-expressing viral vector. Transfection efficiency was found to be > 90% as measured by flow cytometry and immunocytochemistry. The cells were cultured in RPMI 1640 (WelGENE, Inc., Daegu, Korea) containing 10% (vol/vol) heat-inactivated fetal bovine serum (FBS, Gibco^TM^/Life Technologies, Grand Island, NY), penicillin 100 U/mL, and streptomycin 100 μg/mL (Invitrogen^TM^/Life Technologies, Carlsbad, CA) at 37°C/5% CO_2_.

Human BM-derived MSCs (passage 2) were obtained from the Center for the Preparation and Distribution of Adult Stem Cells (http://medicine.tamhsc.edu/irm/msc-distribution.html), which supplies standardized preparations of BM-MSCs under the auspices of an NIH grant (5P40 OD 011050) [29]. The cells consistently differentiated into three lineages in culture. They were negative for hematopoietic markers (CD34, CD36, CD117, and CD45) and positive for mesenchymal markers (CD29 (95%), CD44 (>93%), CD49c (99%), CD49f (>70%), CD59 (>99%), CD90 (>99%), CD105 (>99%), and CD166 (>99%)). The cells were maintained in Complete Culture Medium for MSCs (CCM). The CCM contained alpha-MEM (Gibco^TM^/Life Technologies), 17% (vol/vol) FBS (a lot selected for rapid growth, Atlanta Biologicals, Norcross, GA), 100 U/mL penicillin (Invitrogen^TM^/Life Technologies), and 100 μg/mL streptomycin (Invitrogen^TM^/Life Technologies), and 2 mM L-glutamine (Gibco^TM^/Life Technologies). The cells were passaged at a density of 500 cells/cm^2^ when they reached 70–80% confluence.

### Animal model for lacrimal gland B-cell lymphoma

Animal experiments were performed in strict accordance with the ARVO statement for the use of animals in ophthalmic and vision research. The experimental protocols were approved by the Institutional Animal Care and Use Committee of Seoul National University Hospital Biomedical Research Institute. Eight-week-old female BALB/c mice (H-2^d^) were purchased from Orient Bio Inc. (Seongnam, Korea) and housed in a specific pathogen-free environment.

To establish the lacrimal gland B-cell lymphoma, GFP-expressing A20 cells (A20-GFP, 1 × 10^5^ or 1 × 10^6^/20 μl PBS) were injected into extraorbital or intraorbital lacrimal glands using a Hamilton syringe with a 33-gauge needle (Hamilton, Reno, NV) under an operating microscope (Carl Zeiss, Jena, Germany). The same volume of PBS was injected as negative control. For MSC treatment, 1 × 10^5^ MSCs (1 × 10^5^/10 μl PBS) were mixed with A20-GFP cells (1 × 10^6^/10 μl PBS) (MSCs to tumor cells = 1:10) and injected into lacrimal glands.

### Measurement of lacrimal gland volume and GFP imaging

The extraorbital and intraorbital glands were excised, and the length (L) and width (W) of each gland were measured by calipers. The gland volume was calculated using the following formula: Volume = (L × W^2^)/2. The IVIS^®^ Lumina II System (Perkin Elmer, Waltham, MA) was used to quantify the GFP signal from the excised glands.

### Histopathology

The extraorbital and intraorbital glands were extracted and fixed in optimal cutting temperature compound (Sakura Finetek USA, Inc., Torrance, CA). The glands were preserved at -80°C until sectioning. The sections were cut at 4 μm and subjected to hematoxylin-eosin staining and immunostaining for GFP, CD3, CD11b, and CD19. The primary antibodies used were Alexa Fluor^®^ 488 anti-GFP antibody, anti-mouse Alexa Fluor^®^ 700 CD3 antibody, Alexa Fluor^®^ 594 anti-mouse CD11b antibody, and Alexa Fluor^®^ 647 anti-mouse CD19 antibody (all from BioLegend^®^, San Diego, CA). A mounting medium containing DAPI (VECTASHIELD^®^ Mounting Medium, Vector Laboratories, Inc., Burlingame, CA) was used for counterstaining.

For TUNEL staining, ApopTag^®^ Red in situ Apoptosis Detection Kit (EMD Millipore, Billerica, MA) was used.

To detect MSCs in the glands, the sections were stained with mouse anti-human nuclei (Cy3 conjugate, 1:50) (MAB1281C3, Millipore, Billerica, MA) and mouse anti-human mitochondria (1:50) (MAB1273, Millipore), followed by Alexa Fluor 488 goat anti-mouse IgG (1:500) (A11001, Molecular Probes^®^/Life Technologies, Eugene, OR).

### Flow cytometry

A single-cell suspension was collected by mincing the glands between the frosted ends of two glass slides in RPMI 1640 media (WelGENE, Inc.) containing 10% FBS (Gibco^TM^/Life Technologies), and stained for 30 min at 4°C with the following fluorescence-conjugated anti-mouse antibodies: CD4, CD8, Foxp3, CD11b, CD11c, CD40, Ly6C, Ly6G, or MHC class II (H-2^d^) (all from eBioscience, San Diego, CA). We included FMO (fluorescence minus one) control for each antibody and used as gating control. To exclude dead cells from analysis, all samples were stained with the Fixable Viability Dye eFluor™ 780 (eBioscience). The data were analyzed based on the gating boundaries acquired with each FMO control after excluding FVD^+^ dead cells. For Foxp3 intracellular staining, the Foxp3/Transcription Factor Staining Buffer Set (eBioscience) was used for fixation and permeabilization. The cells were read on S1000EXi Flow Cytometer (Stratedigm, Inc., San Jose, CA), and data were analyzed using Flowjo program (Tree Star, Inc., Ashland, OR).

### Real-time RT-PCR for mouse cytokines

The extra- and intraorbital glands were cut into small pieces, lysed in RNA isolation reagent (RNA Bee; Tel-Test, Friendswood, TX), and homogenized using a sonicator (Ultrasonic Processor, Cole Parmer Instruments, Vernon Hills, IL). Total RNA was extracted using RNeasy Mini kit (Qiagen, Valencia, CA) and used to synthesize first-strand cDNA by reverse transcription (High Capacity RNA-to-cDNA Kit, Applied Biosystems^®^/Life Technologies, Carlsbad, CA). Real-time PCR for mouse *Il1β, Il2, Il4, Il10, Il12a, Ifnγ, Tgfβ1, Tnfα, Arg1,* and *Nos2* was performed using TaqMan Gene Expression Assays (Applied Biosystems) and TaqMan Fast Master Mix (Applied Biosystems). A mouse *Gapdh* was used for normalization of gene expression. All the PCR probe sets (TaqMan Gene Expression Assay kits) were purchased from Applied Biosystems. The assays were performed in duplicates for each biological sample. Calculated delta-Ct values between gene of interest and *Gapdh* were used to obtain relative expression values (2^−ΔΔCt^).

### *In vitro* A20 viability and apoptosis assays

A20 cells were cultured alone or co-cultured with MSCs at a ratio of 10:1 and 1:1 (A20 cells to MSCs) for 2, 4, and 24 h. The non-adherent cells (A20 cells) were collected to assess cell viability and apoptosis. For cell viability, the MTT assay was used (Vybrant^®^ MTT Cell Proliferation Assay Kit; Invitrogen^TM^/Life Technologies). For apoptosis, cells were incubated with a combination of ANX (Molecular Probes, Inc.) and PI (Molecular Probes, Inc., Leiden, The Netherlands), and analyzed by flow cytometry. The populations of early apoptotic cells (ANX^+^PI^−^) and late apoptotic cells (ANX^+^PI^+^) were compared between groups. The experiments were performed four times independently.

### Statistical analysis

Comparisons of values among two groups were performed with the Student’s *t* test using GraphPad Software (GraphPad Prism®, Inc., La Jolla, CA). To compare means of more than two groups, data were analyzed by one-way ANOVA and Tuckey’s Honestly Significant Difference test. Values were shown as the mean value ± SD, and differences were considered significant at *p* < 0.05.

## SUPPLEMENTARY MATERIALS FIGURE


