# Supplementary material for: Mesenchymal stromal cells promote B-cell lymphoma in lacrimal glands by inducing immunosuppressive microenvironment
Source: Oncotarget. 2017 Aug 7;8(39):66281–92. doi: 10.18632/oncotarget.19971 (PMC5630411; doi:10.18632/oncotarget.19971)
Supplement: Supplementary file 1 [file oncotarget-08-66281-s001.pdf]

Mesenchymal stromal cells promote B-cell lymphoma in lacrimal glands by inducing immunosuppressive microenvironment

SUPPLEMENTARY MATERIALS

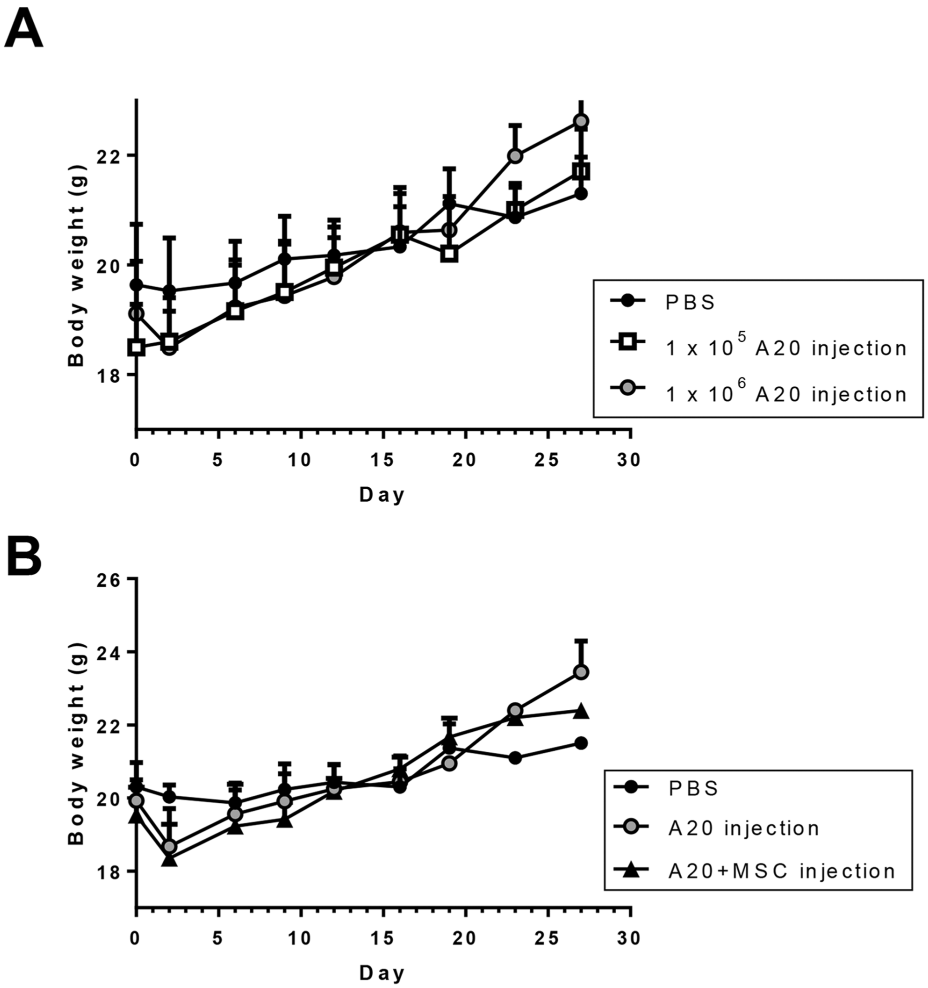

Supplementary Figure 1: (A) Body weight measurements (mean + SD) of BALB/c mice until 4 weeks after injection of PBS, 1×10^5 or 1×10^6 A20 B lymphoma cells into lacrimal glands. **(B)** Time course change in body weight (mean + SD) in the mice receiving PBS, 1×10^6 A20 cells, or A20+MSC cells.

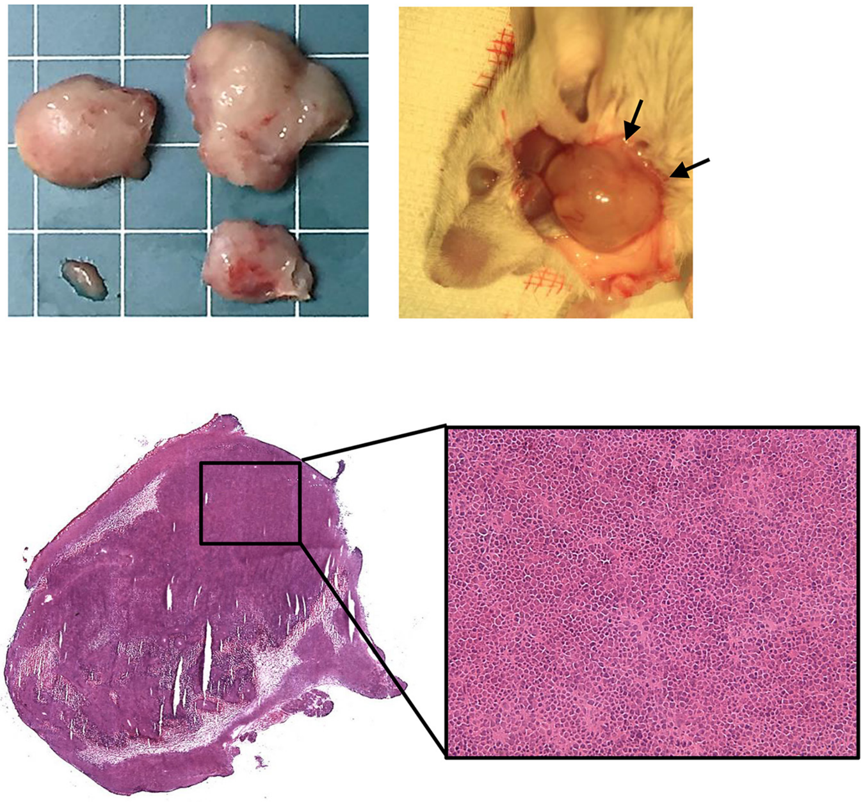

Supplementary Figure 2: Gross and histologic images of extraorbital gland lymphomas at 4 weeks after 1 × 10^6 A20 B lymphoma cell injection.

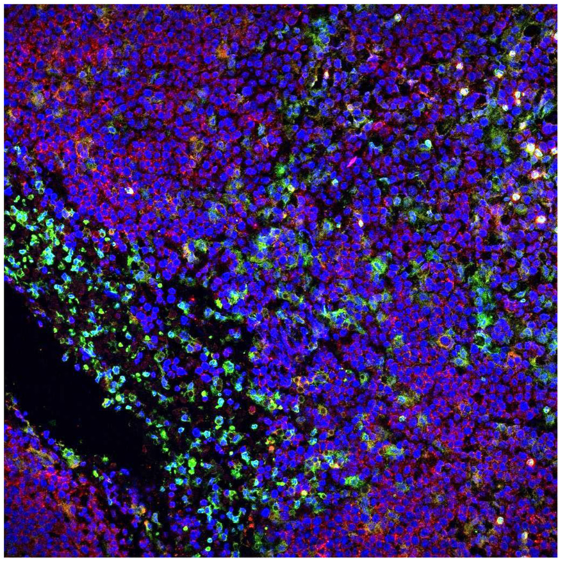

Supplementary Figure 3: $CD11b^+$ cells infiltrating the extraorbital lacrimal gland are in close contact with $CD19^+$ tumor cells. Original magnification × 200.

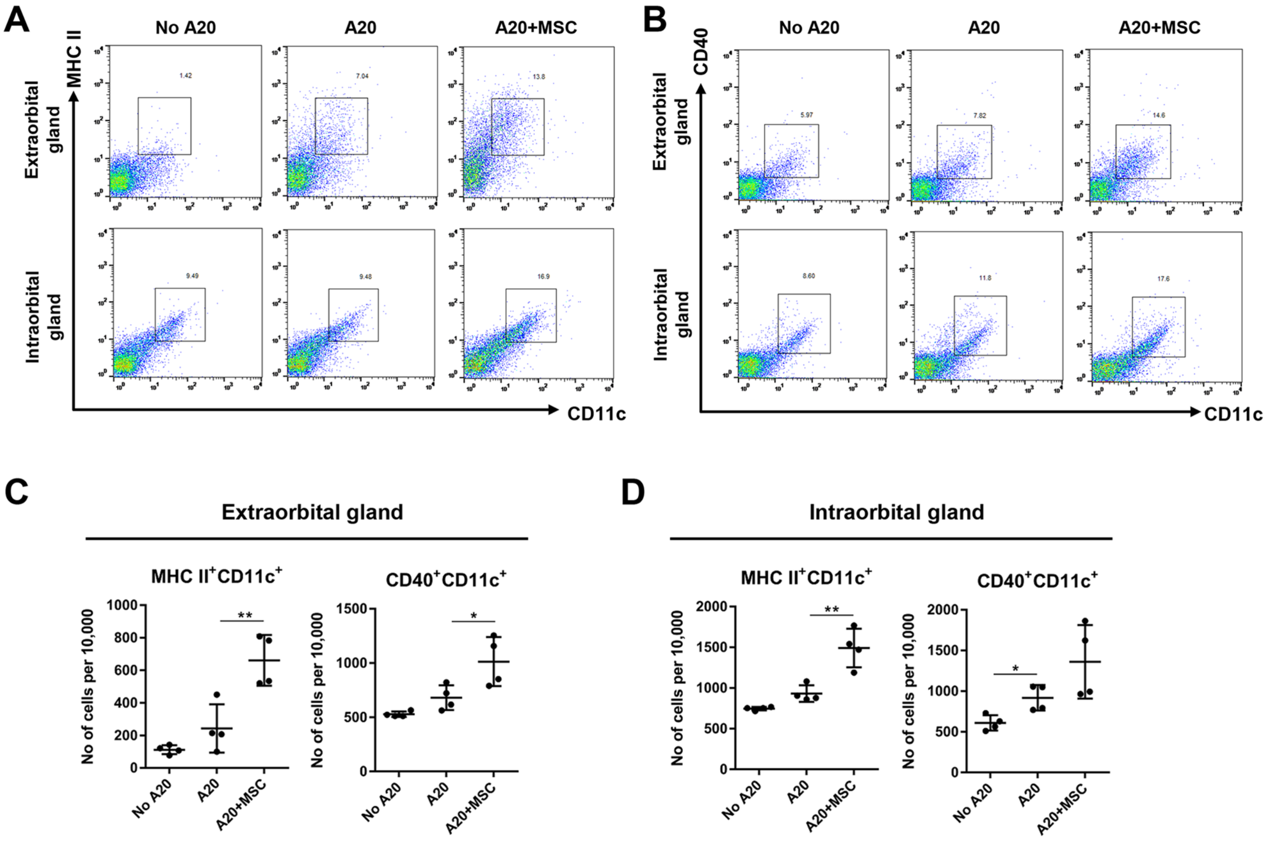

Supplementary Figure 4: Representative results for **(A)** MHC II$^+$CD11c$^+$ and **(B)** CD40$^+$CD11c$^+$ cells in extraorbital and intraorbital lacrimal glands at 1 and 2 weeks after either 1 × 10^6 A20 cell injection or A20+MSC co-injection. PBS was injected in control group (No A20). MSCs increase the number of MHC II+CD11c+ dendritic cells (DCs) and most of DCs are CD40+CD11c+ cells in lymphoma-bearing **(C)** extraorbital and **(D)** intraorbital lacrimal glands. FMO (fluorescence minus one) control per each antibody was used as gating control, and the analysis was performed after excluding dead cells with FVD (Fixable Viability Dye) staining. Dot indicates a single animal, and the bar indicates the mean ± SD. * $p < 0.05$, **$p < 0.01$.

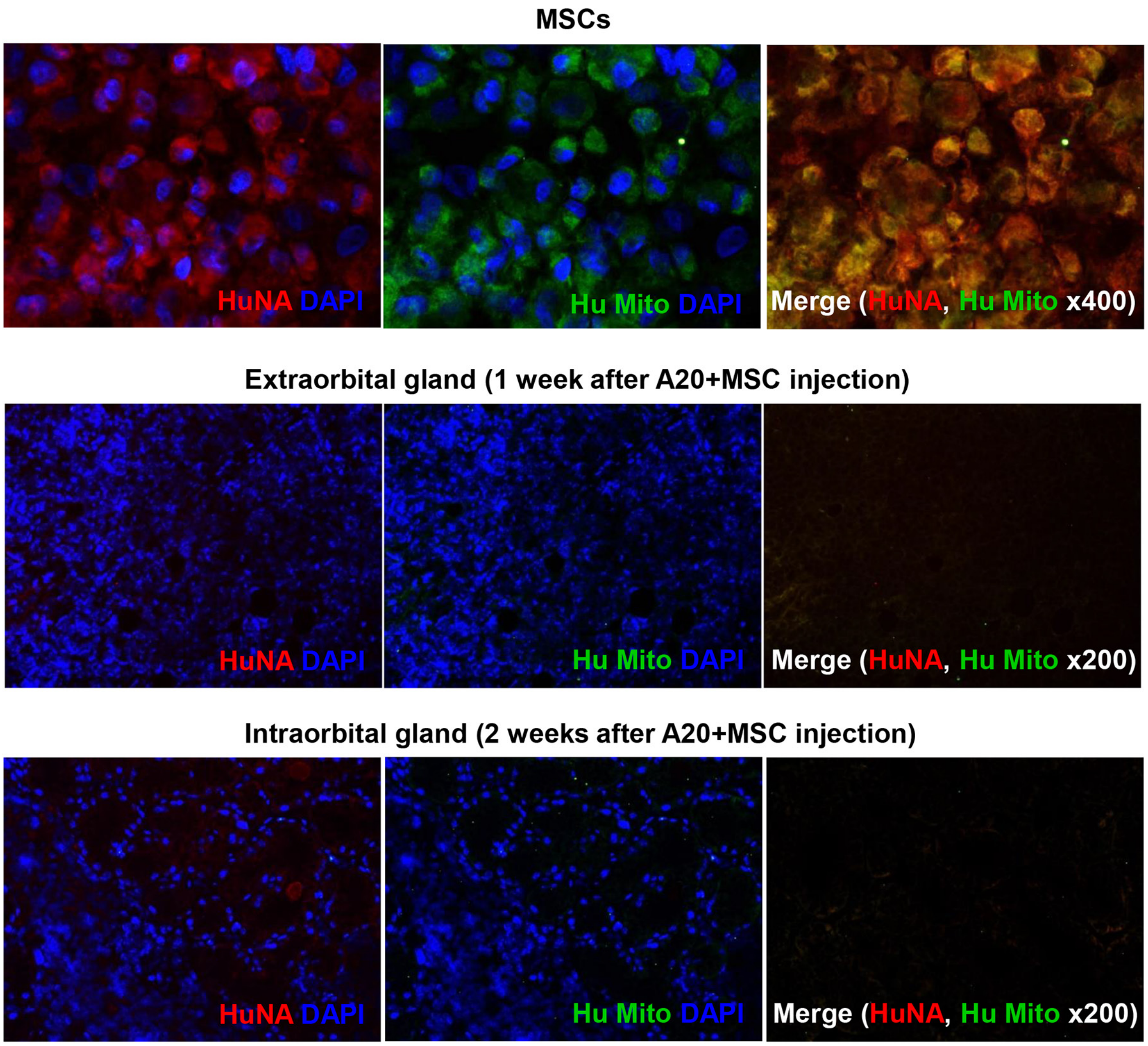

Supplementary Figure 5: Immunostaining of mouse lacrimal glands with human-specific mitochondria and nuclear antigen at 1 and 2 weeks after A20+MSC co-injection. Original magnification × 400. *in vitro* cultures of MSCs were stained as positive controls. Human mitochondria (Hu Mito) were stained in green and human nuclear antigens (HuNA) in red. The nuclei were stained with DAPI (blue).
